# Public perception and awareness of waste management from Benin City

**DOI:** 10.1038/s41598-020-79688-y

**Published:** 2021-01-11

**Authors:** P. O. Adekola, F. O. Iyalomhe, A. Paczoski, S. T. Abebe, B. Pawłowska, M. Bąk, G. T. Cirella

**Affiliations:** 1 Demography and Social Statistics Programme, Department of Economics and Development Studies, College of Management and Social Sciences, Covenant University, Ota, 112233 Nigeria; 2 Centre for Economic Policy and Development Research, Covenant University, Ota, 112233 Nigeria; 3 Polo Centre of Sustainability, 18100 Imperia, Italy; 4 Ministry of Environment and Sustainability, Edo State Government, Benin City, 300372 Nigeria; 5 Department of Environmental Sciences, National Open University of Nigeria, Abuja, 900211 Nigeria; 6 Faculty of Economics, University of Gdansk, 81-824 Sopot, Poland

**Keywords:** Climate-change mitigation, Sustainability, Risk factors, Climate-change mitigation

## Abstract

Poor waste management is increasingly becoming a major challenge for municipalities, globally. Unlike previous waste management studies in Nigeria, this study examines the implications of waste management to regional greenhouse gas emissions based on awareness levels and perception of urban inhabitants. Benin City was divided into four residential zones: core, intermediate, suburban, and planned estates. Blocking was utilized to collect data from a total of 2720 randomly selected inhabitants through a self-administered survey. Results reveals low awareness level in terms of indiscrimination dumping of waste, thereby promoting sustainable mitigation and adaptation measures region-wide. It is imperative to integrate various aspects of regional government services such as infrastructure, urban planning and development, socioeconomics, public health, and regulation enforcement. Waste management policy is strengthened via working groups, community, and regional authorities.

## Introduction

Globally the management of solid waste is an enormous challenge for municipalities. Previous studies indicate increase in population with relating concomitant urbanization dynamics and growth of conurbations will further exacerbate this challenge^1–6^. In 2006, Monni et al.^7^ reported on five global scenarios compiled from 1990 to 2050 on global post-consumer waste generation. They stated global emissions from landfills in 1990 to be 340 Tg CO_2_ eq with an increase to 1500 Tg CO_2_ eq by 2030 and to 2900 Tg CO_2_ eq by 2050. Laurent et al.^8^ and Zhang et al.^9^ more recently reviewed greenhouse gas (GHG) emissions from landfills and listed a number of important mitigation methods as well as development and dissemination of updated knowledge-based frameworks. At present, the World Bank affirms 2.01 billion metric tons of municipal solid waste (MSW) are produced annually worldwide, with estimates reaching 3.40 billion metric tons by 2050^10^. Current worldwide estimates indicate 13.5% of waste is recycled and 5.5% is composted, with an estimated one-third and 40% of MSW as not managed properly and instead dumped or openly burned^11^. This mismanagement is significantly split between developed and developing countries. For example, about 80% of solid waste in African countries is dumped indiscriminately in open spaces, streets, stormwater drains, rivers, and streams^12^, thereby, estimated to contribute to about 29% of the global GHG emissions and expected to increase to 64% by 2030^7^. This is largely due to uncontrolled population growth and affluence, unsustainable development activities, and expansion of waste collection services without sufficient management strategies (i.e., changes from dumpsites to sanitary landfills with a lack of landfill gas collection). Dladla et al.^13^ identified factors associated with indiscriminate dumping of waste in eleven African countries and found institutional weaknesses and lack of awareness as key starting points to the problem. Lack of public perception can be a limitation to what can be done and, ultimately, to what can be achieved^5,14–19^. African countries that are heavily inundated with indiscriminate MSW constitute health challenges to residents of the area as GHGs are released^8,9^. Institutional weaknesses may occur in the form of ambiguity of waste management policy and legislation since MSW activities effect the core infrastructural framework of a municipality and its residents. Hence, increasing awareness of inhabitants may have a positive impact on attitude as well as perception towards the environment.

Nigerian cities are largely characterized with having solid waste disposal problems. They are typified by overflowing dumpsters, mountains of open refuse dumps (i.e., on virtually every street), and makeshift landfills on the edge of larger suburbs and towns. Attendee problems are also evident, especially where burning occurs, since properly operated landfills are nonexistent and often rodent infested with surface and ground water pollution concerns^4,12,20–23^. As such, the collection and transport of MSW requires the largest demand on municipal budgets but has been seen to have the greatest impact on urban living^24^. Oseghale^25^ and Ezeudu and Ezeudu^26^ reported that in Nigeria 68% of MSW is indiscriminately dumped, 20.8% is disposed through inappropriate landfill sites, and 10.7% is burnt. According to the GHG emissions inventory from 2000 to 2018 the contribution from inappropriate waste was 3.0%^27^, which is expected to double within the next five years, considering that waste generation rate is estimated between 0.5 and 1.0 kg/person/day^25^; however, this is subject to change due to unplanned population growth and unsustainable urban waste management practices Nigeria. Moreover, Ossai^28^ illustrated the complexity of waste composition Nigeria, stating biodegradable waste accounts for about 50% of annual MSW with less than 10% of waste as manageable.

Indiscriminate dumping of waste has environmental, public health, and economic impacts in most cities^29,30^. It can result in contaminated water and bio-chemical poisoning of food supplies^31^ as well as contribute to GHG emissions^32^. Aliu et al.^4^ underscored that MSW management problems in Nigeria have been attributed to lack of awareness, lack of enabling legislation, inappropriate technology, poor infrastructural maintenance as well as noncommittal posture of waste management workers, group behavior, education, poverty, and corruption. In Nigeria, managing MSW is one of the most costly urban services, absorbing up to 1% of gross domestic product and 40% of municipal revenue^24,27^. Benin City, the largest urban center in Edo State, in southern Nigeria houses much of the challenges, thus far, described. Many of its suburbs (i.e., including residential areas and public places) are littered with domestic and sewage waste, garbage, and chemical waste. Industrial operation is characterized by the generation of large volume of MSW in the form of solid, liquid and gas—some toxic. As a result, the Edo State Waste Management Board (EWMB), established by the local authorities, put in place a monitoring program to regulate environmental quality and implement steps towards a waste-free society. Despite its efforts, Benin City still falls short of achieving Board benchmark levels and ongoing waste management practices are needed. This paper examines an in-depth look at the level of awareness and attitude of its inhabitants towards MSW disposal and possible contribution to regional GHG emissions. The research is in accordance with the local government, with the intention of enhancing MSW management programs for other Nigerian cities and beyond which face similar problems.

## Results

### Household socio-demographic characteristics

Household results examined demographic characteristics (i.e., gender, age, occupation, educational status, marital status of the respondents, and awareness of GHG emissions effect on MSW) and provided useful insight into the socioeconomic characteristics of city. Out of the total sample of interviewed respondents, 58.3% were male while 41.7% were female. In terms of gender differences, Wu et al.^33^ disclosed that gender, family size, household income, and educational level have statistically significant impacts on household GHG emission levels. Moreover, they concluded that relationship status and family size did not positively effect GHG emissions output, while family income and educational level did not negatively affect it. Correlatively, Jaiswal and Shah^34^ showed that households did not differ significantly in terms of carbon footprint due to gender, age, education, and family income. Utilizing these two studies, our study revealed similar household results. Interestingly, awareness that GHGs are emitted via MSW was split almost evenly with 51.7% as not aware and 48.3% as aware (Table 1).

**Table 1:** Survey variable results for Benin City.

Variable	Category	2009^a^ (%)	2019 (%)	${x}^{2}$
Gender	Male	72.90	58.30	0.003***
	Female	27.10	41.70	
Age	≤ 20	0.00	6.70	0.001***
	21–35	29.63	33.30	
	36–45	29.07	22.50	
	46–55	27.80	29.20	
	≥ 56	13.50	8.30	
Primary occupation	Traders	21.20	33.30	0.01**
	Farmer/fisherman	6.38	4.20	
	Civil servant	15.08	25.00	
	Private professional	19.05	25.00	
	Other^b^	38.29	12.5	
Education level	GCE/SSCE/WAEC	12.78	12.50	0.045*
	NCE/ND	30.55	29.20	
Awareness of GHG emissions effect on MSW	Not aware	Not available	51.70	0.001***
	Aware	Not available	48.3	

*GCE* General Certificate of Education, *SSCE* Senior Secondary Certificate Examination, *WAEC* West African Examination Council, *NCE* National Certificate of Education, *ND* National Diploma.

^a^Data sourced from Ekhaese et al.^35^; significance level of each variable: *p = 0.1, **p = 0.05, **p = 0.01.

^b^Other = students, retired persons, and unemployed.

In 2019, the household carbon footprint clearly has had an economic change city-wide. This change is correlated (i.e., significantly) with increased employment levels from the decade earlier. It can be noted, as professional skills improved (i.e., in terms of higher levels of primary occupation), the level of socioeconomic status in Benin City has grown and offered a better standard of living. Utilizing observational data, i.e., provided by the EWMB, as well as our own qualitative notes taken during fieldwork, it can be deduced that in Benin City higher levels of affluence are directly correlated with increased GHG emissions, i.e., via increased levels of MSW. Our 2019 results also pinpoint important significance levels in terms of gender and age—both demonstrating a strong survey sample and wider reach of the population—versus Ekhaese et al.’s^35^ findings from 2009.

### Perception of waste management and its contribution to GHG emissions

Unarguably, GHG emissions have become an important issue related to waste management in recent years; however, in developing countries many factors are associated with indiscriminate dumping of MSW—e.g., such as in Nigeria. Results from the survey explicitly focused on trying to understand awareness levels of Benin City’s inhabitants in terms of potential repercussion of indiscriminate dumping of MSW to the environment. The majority, i.e., 62.6%, reported their awareness level as very low. Only 8.3% of the respondents stated it as very high (Table 2). Since inhabitants did not perceive indiscriminate dumping of solid waste as a significant issue, it can be inferred they are not likely to improve their waste disposal and management practices. Correspondingly, Dladla et.al^13^ examined this issue by reviewing 40 journal MSW-related articles and, interestingly, linked institutional weakness and lack of awareness as not among the leading factors, however, when combined these factors deepened the challenge of proper MSW management. Likewise, the majority of survey respondents mentioned they had some notion that GHG emissions were problematic but lacked knowhow that indiscriminate MSW could augment the problem. This lack of awareness ties in with previous research by Yoada et al.^36^ where they conducted research in Nigeria that showed perception of domestic waste disposal, in terms of community attitude about and perception of sanitation, interlinked with waste management concerns. This overlap is encouraging as it establishes a precedent for awareness-based GHG emissions for stakeholders and communities confronting this issue.

**Table 2:** Awareness level in Benin City of the repercussions of indiscriminate waste disposal to the environment.

Likert answer—category	Respondents	Percentage (%)
1—very high	226	8.3
2—high	565	20.8
3—low	226	8.3
4—very low	1703	62.6

**Figure 1:**
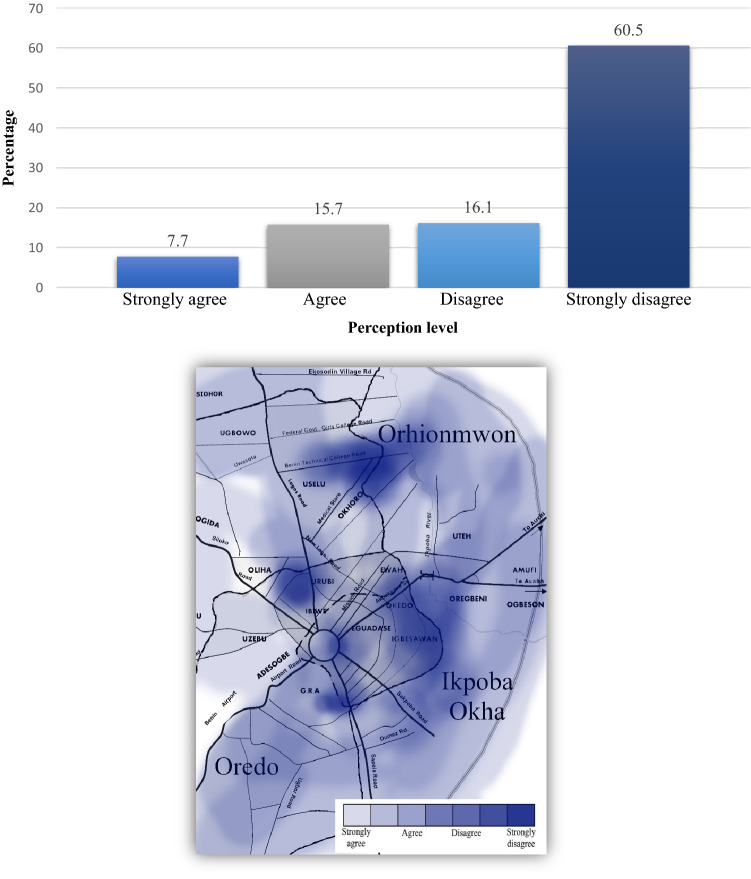
Perception of whether GHG emission techniques and methods used by the government adequately create awareness about the health implications of MSW in Benin City: (top) graph, (bottom) mapped results.

In terms of GHG emission techniques and methods used by Benin City’s government authority, the survey examined the adequacy of whether it was properly informing citizens of the direct health implications from MSW. Health concerns from indiscriminate dumping of waste throughout the city have been on the rise in recent years^27^. This troubling fact confers with our results that the majority of inhabitants city-wide strongly disagreed with any measures being implemented or properly being disseminated to the public at large. Overwhelmingly, results showed that 60.5% of the respondents did not feel they were properly informed on the issue and voiced concern that a lot more needed to be done. Of the 7.7% of persons that strongly agreed, secondary data (i.e., via qualitative discussion with respondents) found that many of them had either tertiary education, higher social status (i.e., in terms of work), and tended to be more tech savvy. Clearly, these manifested limitations by the local government, Federal Ministry of Environment, and EWMB raise a lot of red flags. These agencies should immediately implement better public awareness and promote understanding of the essential linkage between the environment and development^37,38^. Complementarily, an educative program could inform the city’s citizenry of the GHG emission techniques and methods the government is using to better public health awareness and improve public confidence (Fig. 1).

**Figure 2:**
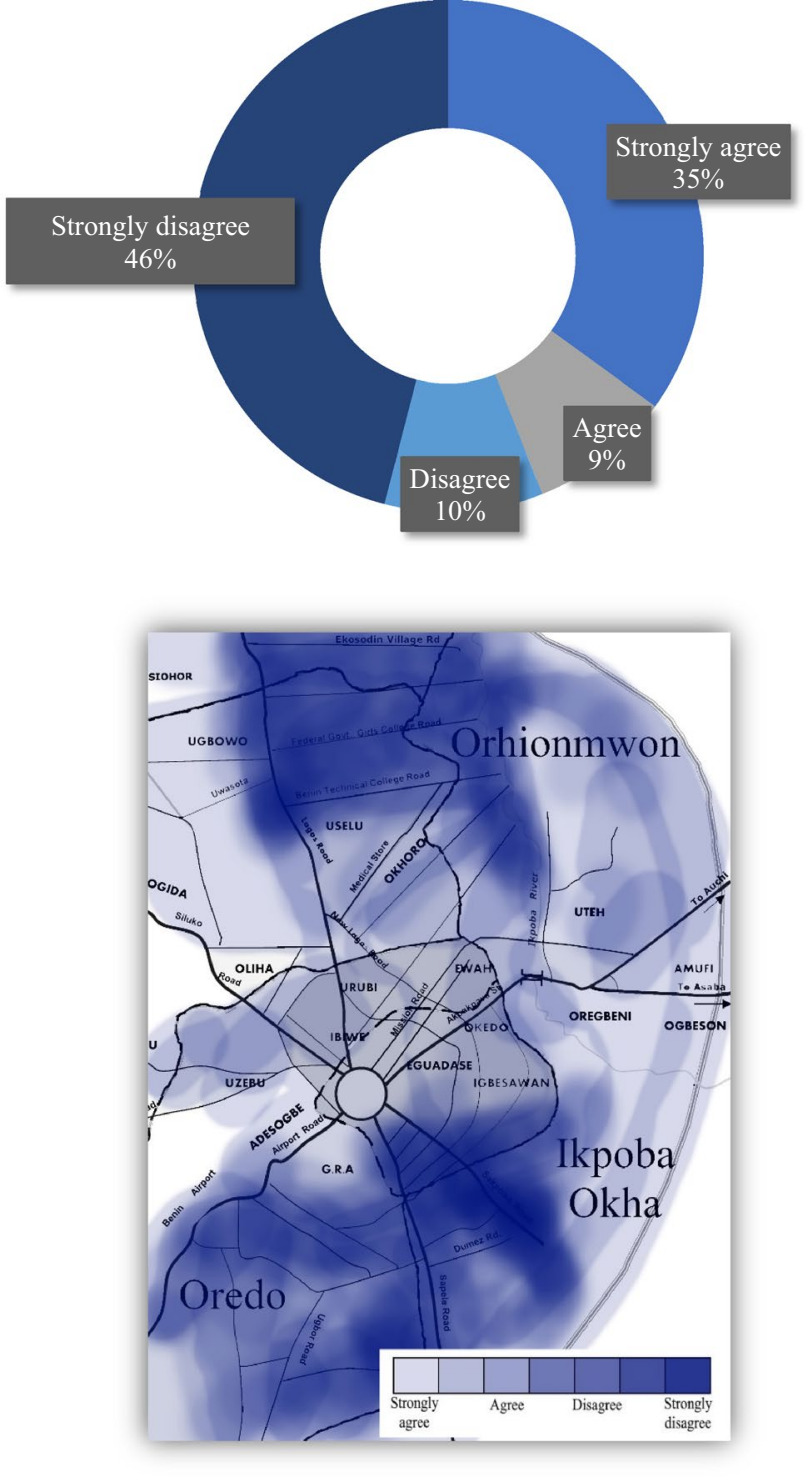
Perception that there is growing threat level from MSW disposal in Benin City: (top) graph, (bottom) mapped results.

**Figure 3:**
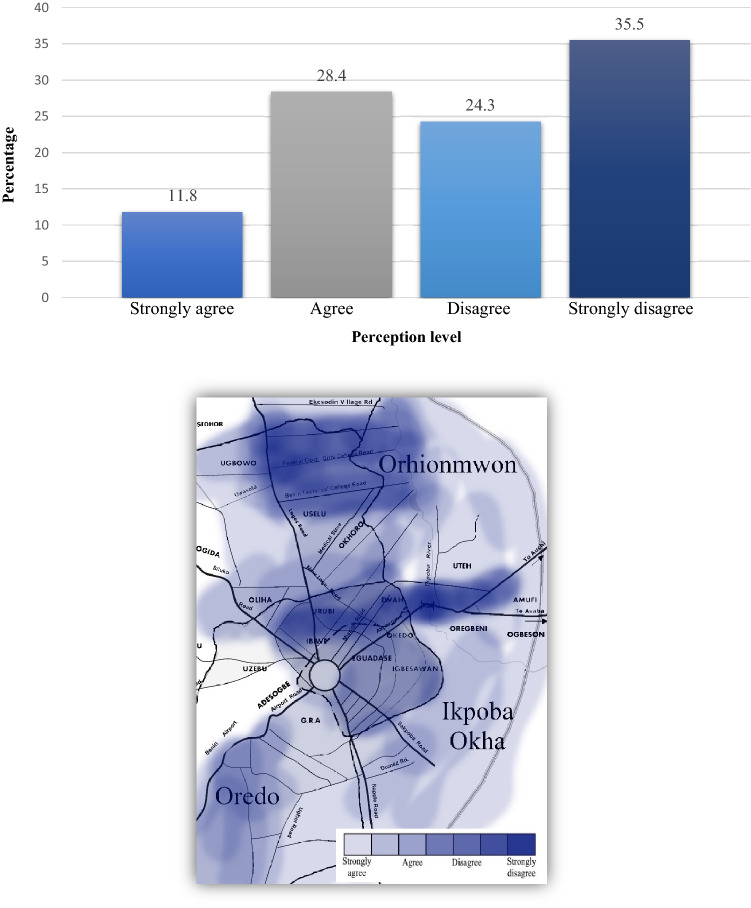
Perception that waste management can reduce the global effect of GHG emissions: (top) graph, (bottom) mapped results.

In a related question to the awareness of the repercussions of indiscriminate waste disposal to the environment, a supplementary question asked whether they believed MSW disposal was growing towards a threatening level. This question identified the threat level of the populace and offered insight into local knowledge of the problem as well as other inferred factors such as level of livability, acceptability, and advocacy to change under current conditions. Results showed slightly more than a third of respondents (i.e., 35%) strongly agreed that growing domestic waste disposal was or already is a threat to their livelihood (Fig. 2). According to Yoada et al.^36^, comparative findings from Ghana showed once threat levels augment domestic and household activities in urban environments have a high potential to serve as breeding grounds for rodents and insects causing an increased “risk of the spread of parasitic and zoonotic diseases”^39^. Moreover, debris indiscriminately disposed throughout the streets can choke drains and block waterways—creating the increased possibility of flooding during the wet season^40^. Similarly, we examined whether there was the belief that waste management could be used to reduce the global effect of GHG emissions (Fig. 3), touching upon the concept of climate migration and its urgency. For example, the matter of properly preparing for flood events—something that Nigerians, and especially, inhabitants from Edo State clearly understand^41^—could be used to leverage waste management matters with the recent memory of flood devastation that has cost the country enormous damage and harm. Unfortunately, more than half of the respondents from Benin City either disagreed or strongly disagreed that waste management could help reduce the global effect of GHG emissions. This directly correlates with the previous argument that a campaign-type program should be implemented to help inform the public of the urgency and need to better cognize the importance of MSW disposal and its proper management—for residents and as global citizens.

**Figure 4:**
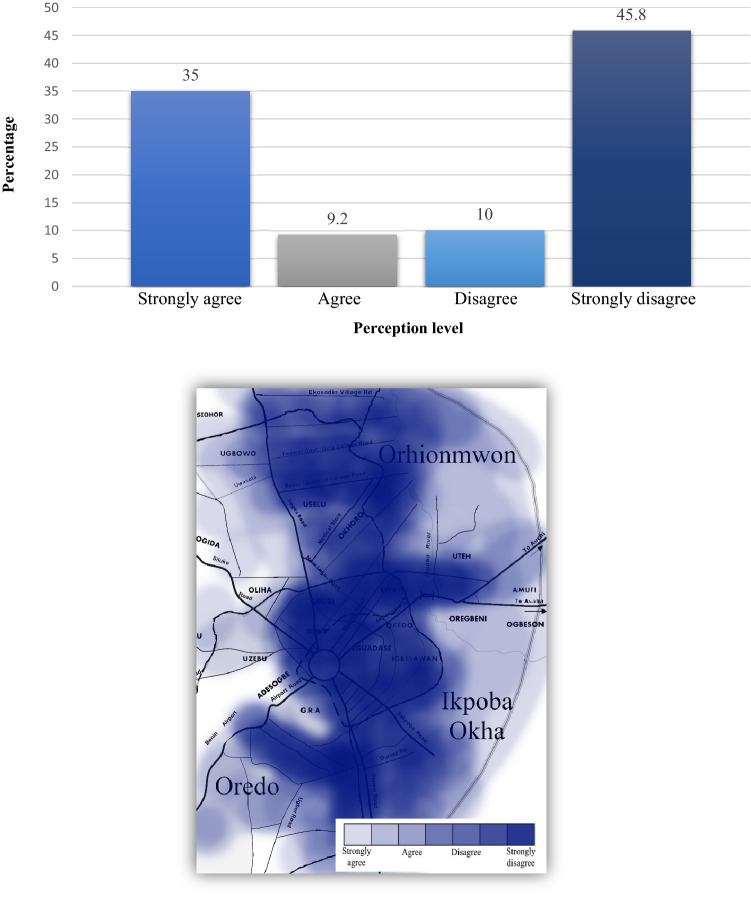
Perception of efficiency level of urban waste control and management in Benin City: (top) graph, (bottom) mapped results.

Another important element of the study examined the perception level of urban waste control and management efficiency. Efficiency is related to the insensitivity of implementing relevant policies, i.e., to find high-efficiency policy routes as well as MSW classification levels adaptable to the municipality in question^42^. As such, cost efficiency of MSW control and management should include: (1) collection and processing of household waste fractions, (2) robustifying measurement errors in municipalities, and (3) continual monitoring of operating environments (e.g., demography and median income)^43,44^. Of particular importance to Benin City, where demand for MSW services is increasing, management efficiency must be a top priority. According to Albores et al.^45^, model development of integrated MSW management should attempt to reduce waste directly at the source “before it enters the chain of waste stream, reuse wastes [as much as possible] for recovery by recycling, and dispose of it through environmentally sound” facilities. To meet the needs of Benin City, urban waste control and management will need to introduce better baseline measures since more than half of the respondents (i.e., 10% disagreed and 45.8% strongly disagreed) with existing measures (Fig. 4). As a starting point and on a positive note, 35% of the respondents stated a strong belief that the level of urban waste control and management in the city was efficient—meaning more than a third of the total respondents were aware of the ongoing measures and of its capacity to fulfil its duties. This result corroborates similar findings by Aliu et al.^4^ in which the role of public–private partnership performance in waste services was understood to be functional by half of the inhabitants of Lagos State.

**Figure 5:**
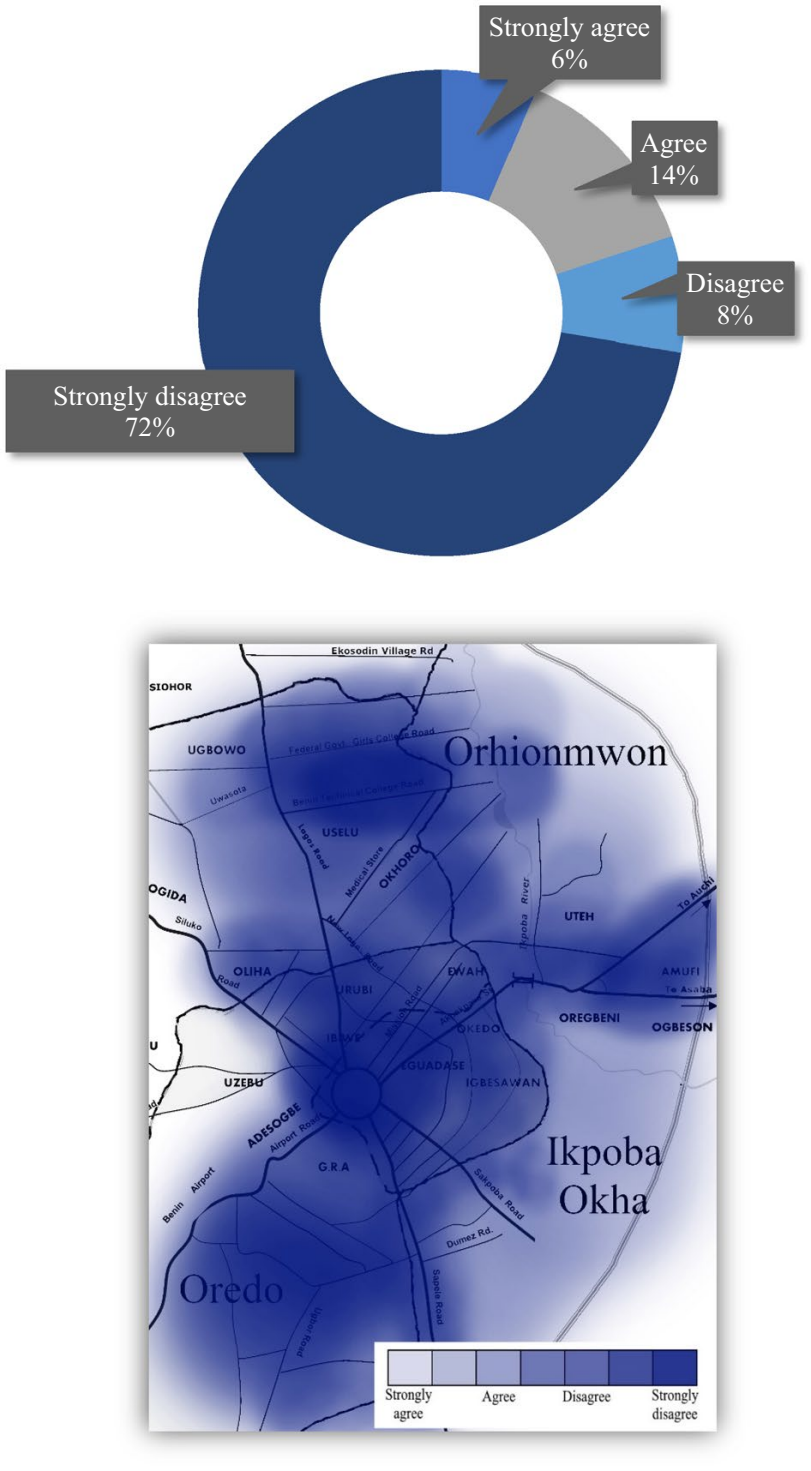
Perception that the authorities responsible for the GHG emissions have a sound knowledge of good practice: (top) graph, (bottom) mapped results.

In reflection of government action to formulate policies and measures to reduce GHG emissions as well as to introduce an informative platform that is responsible and has the backing of the community two questions were asked of respondents. First, awareness level of the government to formulate policies and measures to reduce GHG emissions was collected and tested for significance level using the chi-squared test—total responses were all statistically significant. The majority of respondents stated a very low level of awareness (i.e., 45.8%), followed immediately by a very high level (35%). This contrasting difference can be seen in Table 3 and signals a recurring theme of the stratification of two concurrent societies living side-by-side—one that is more affluent and educated and the other opposite. This is reflective of the world history of development, i.e., developed countries encountered their resource and environmental challenges in stages over the course of 200 years of industrialization, while developing countries are confronting them contemporarily all at the same time^46^. The social stratification of Benin City, unfortunately, is a painstaking reality of a municipality in the developing world that must consider how to implement and formulate best practices at reducing GHG emissions while still addressing the issue of energy conservation and pollution control. Government action should parallel a climate resilient green economy as outlined by Luken and Clarence-Smith^47^. Second, competence and expertise in the field should not only be technically (and scientifically) sound but also parallel the international community and incorporate world bodies when appropriate^48^. Benin City’s respondents overwhelmingly stated that belief in the authorities’ knowledge and good practice of GHG emissions was very low at 72% (Fig. 5). Recent research conducted in Ethiopia by the Ethiopian National Cleaner Production Centre and the Ethiopia Climate Innovation Centre detail that well-documented, feasible, and time-bound strategy for reducing GHG emissions must be grassroots and supported by the industrial sector as well as properly monitored^47^. After closely reviewing these advances we believe Benin City would be well-informed to follow these developments.

**Table 3:** Awareness that the government has formulated policies and measures to reduce GHG emissions.

Likert answer—category	Respondents	Percentage (%)	${x}^{2}$
1—very high	952	35	0.003***
2—high	250	9.2	0.02*
3—low	272	10	0.017**
4—very low	1246	45.8	0.001***

Significance level of each variable: *p = 0.1, **p = 0.05, ***p = 0.01.

Further inference of the results shows the level of knowledge of GHG emissions and MSW among the residents of the various districts in terms of variation streamlined on socioeconomic strata. Residents of Eguadase in Ikpoba Okha as well as Uselu and Okhoro in Orhionmwon have high socioeconomic status and live in planned estates but unfortunately have low knowledge of GHG emissions and danger of indiscriminate dumping of MSW. Hence, they can be blocked together, in terms of sampling, along with other suburban and intermediate areas in Orhionmwon and Oredo who share similarly low knowledge in the variables studied. In contrast, residents of GRA in Oredo who also live in planned estates and belong to the highest socioeconomic strata in the city have high knowledge of GHG emissions and danger of indiscriminate dumping of MSW and were grouped alongside Oregbeni, Ogbeson, and Uteh residents of Oredo and Orhionmwon, respectively (Table 4).

**Table 4:** Randomized block design of the study area.

District	Blocking of sampled area		Randomized and experimental knowledge of GHG emissions
Ikpoba Okha	i. Eguadase	Core, planned estate	LKGHG—DIDMSW
	ii. Oregbeni	Suburban	HKGHG—DIDMSW
	iii. Ogbeson	Planned estate	
Oredo	ii. GRA	Core, planned estate	HKGHG—DIDMSW
	ii. Oko	Intermediate	LKGHG—DIDMSW
	iii. Adesogbe	Suburban	
Orhionmwon	i. Uselu	Core, planned estate	LKGHG—DIDMSW
	ii. Okhoro	Core, planned estate	
	iii. Uteh	Suburban	HKGHG—DIDMSW

**Figure 6:**
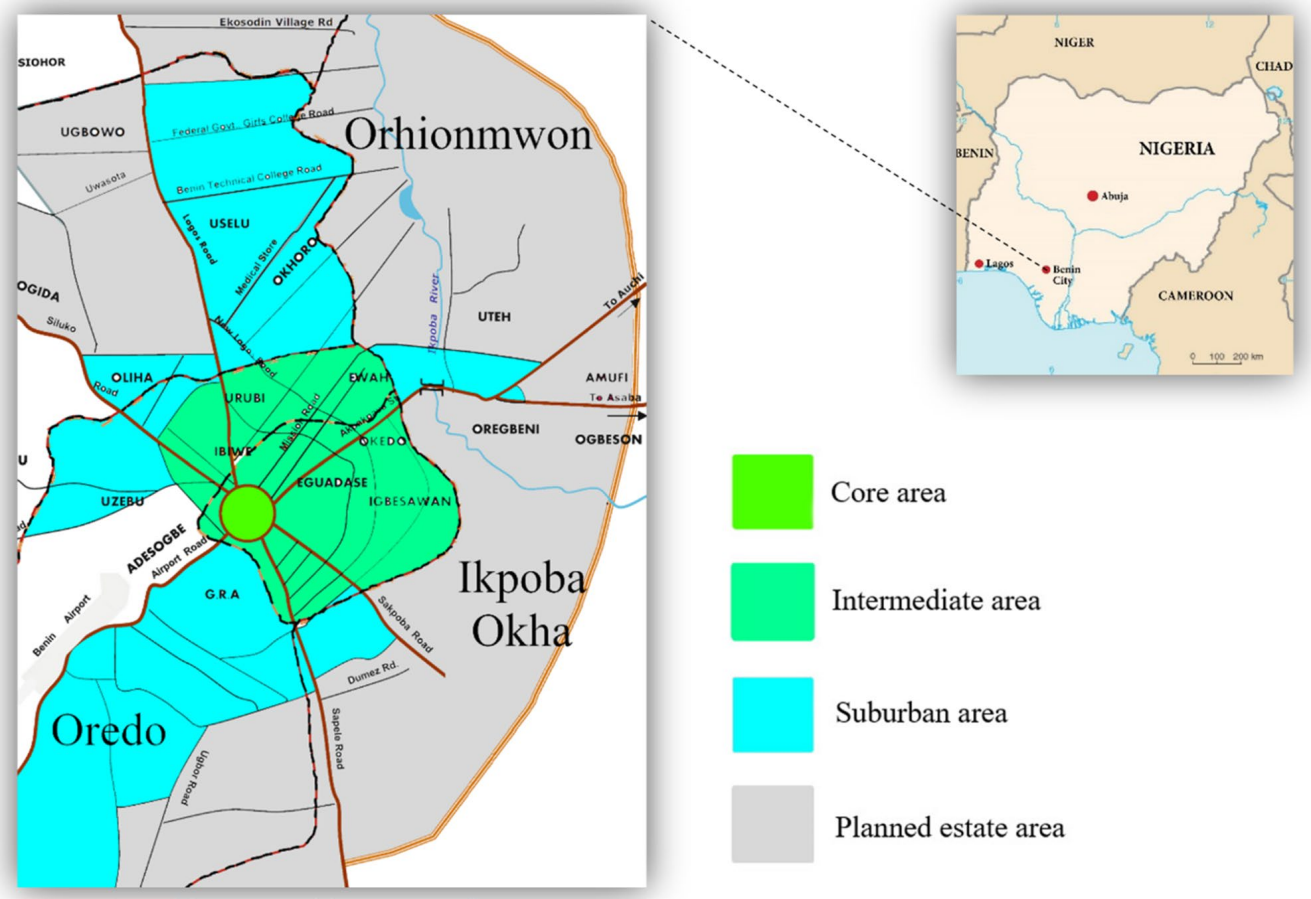
Four sampling areas of Benin City, Edo State, Nigeria.

Extrapolated from Fig. 6 in which inference was made on the knowledge of GHG emissions and MSW based on socioeconomic characteristics of residents from different residential areas.

LKGHG—DIDMSW: Low knowledge of GHG emissions and danger of indiscriminate dumping of MSW, HKGHG—DIDMSW: High knowledge of GHG emissions and danger of indiscriminate dumping of MSW.

## Discussion

Although most developing countries, especially in Africa, are generally assumed to be responsible for an insignificant level of GHG emissions, current increases in population, economic development, and urbanization—paralleled with waste generation and improper management—could result in an enormous increase in emissions levels. We believe this will be the case in Nigeria with respect to MSW which is expected to grow as the population is estimated to increase by as much as 50% in the next 30 years^49,50^. Coupled with the adoption of a Western lifestyle of consumerism, Nigerian urban areas will continue to face a growing MSW trend. This issue is made more significant by poor MSW management and lack of data, especially on actual solid waste generated.

Using cross-sectional data collected from 2,720 randomly selected respondents in Benin City, we underscored the low level of awareness and its possible consequences at the municipality level. As such, sustainable mitigation and adaptation measures would be more successful if they are well understood and enabled people to engage in economic activities that provided a higher standard of living. For instance, adapting recycling as both a mitigation and adaptation option could greatly assist with the current situation since it directly can create local wealth as well as significantly reduce the amount of MSW generating harmful GHG emissions^12,20,26^. A way forward would be to enact an all-inclusive waste management policy to deliver effective awareness and strengthen communication channels between working groups compiling GHG emissions inventory. These groups would also need to provision MSW-related information and awareness within local municipalities by reviewing, bolstering, and building upon existing communication channels through regional authorities. From the initial fieldwork and after recently re-visiting parts of the city as well as re-interacting with some of the respondents the concept of GHG emission knowledge was transferred. However, this should be considered a qualitative observation and would require further investigation to properly establish an educative-based linkage. This viewpoint reflects Amasuomo et al.^51^ and Rahardyan et al.^52^ research that stressed the more the public are informed on waste management matters, the better their perception and disposition to participate in municipal-level waste management. In addition, a recycling approach to sustainable waste management should be incorporated into the formal waste management structure, to provide safer, healthier working conditions so that current scavengers on uncontrolled dumpsites is limited and regulated.

At the national level, the Federal Ministry of Environment^27^ and the Department of Climate Change^53^ are encouraged to enhance the quality of GHG emissions reporting by streamlining data collection, conducting a review of the activity database and its inventory, and identifying opportunities for enhancement. Best practices should be shared top-down to assist with training, activity data sourcing, and filling in gaps that pinpoint changes via quality checks. If outsourcing of the review is not possible, then either the Ministry or Department, respectively, could conduct an internal review using the training manual “Good Practice Study on GHG-Inventories for the Waste Sector in Non-Annex I Countries”^54^—as explained by Santalla et al.^54^ for Argentina. It will be crucial the regional authorities, responsible for data management and aggregation as well as communication between national and local levels of government, to be regularly updated with correct and proper data privileges. Ideally, meetings should be held in-person with representatives of regional authorities to actively engage and facilitate data flows.

## Methods

### Study area

**Figure 7:**
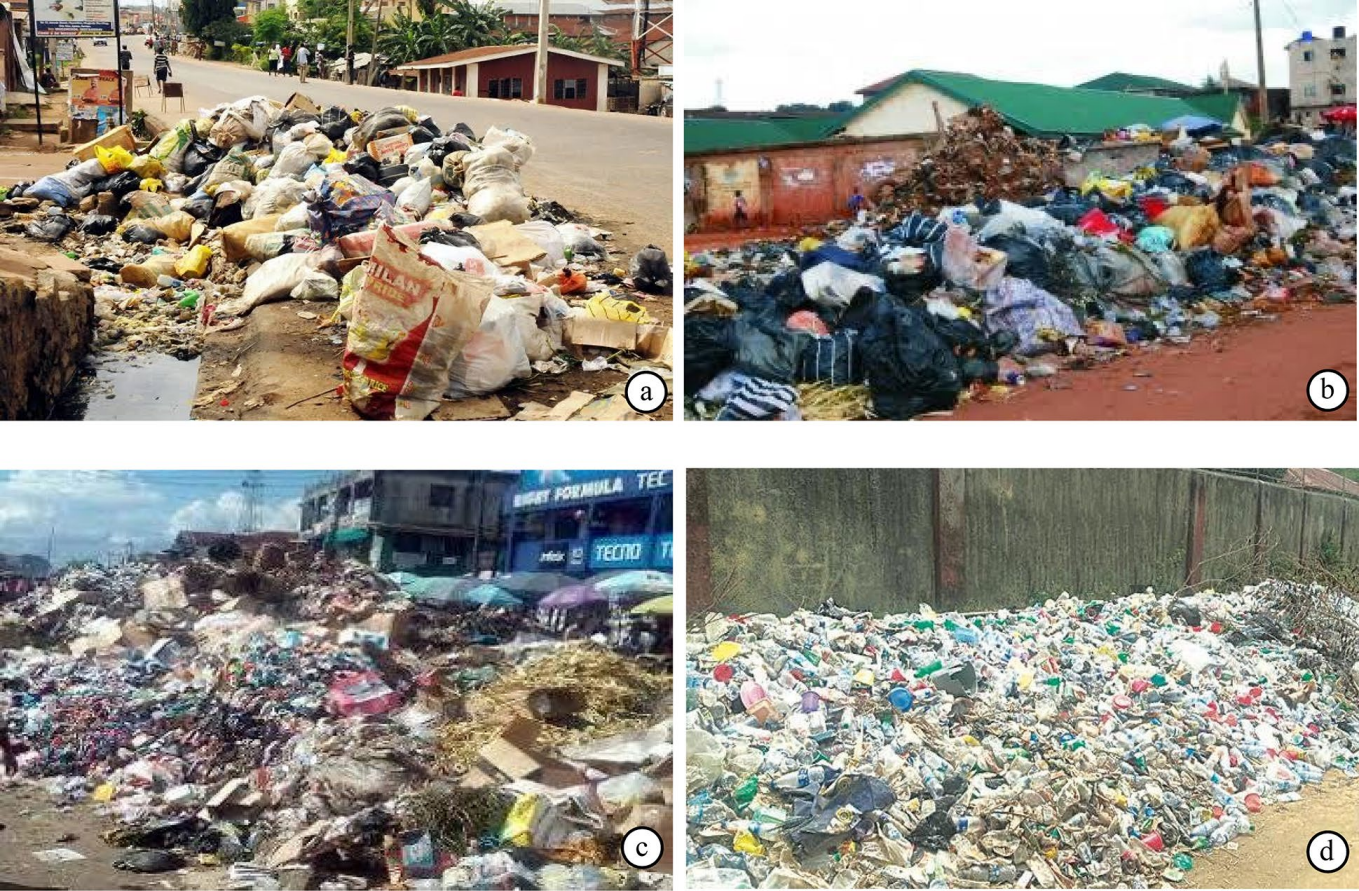
Benin City MSW street-side dumping (**a**,**b**) and makeshift landfills (**c**,**d**); photographs taken by F. O. Iyalomhe on 10 May 2020.

Benin City, Nigeria, with total area of 1,204 km^2^ has a population of 1.72 million (Fig. 6)^55^. Organic and inorganic waste is mostly generated from household food remnants and other biodegradable material commonly found in MSW. Residents habitually dump their household waste in open refuse dumps on virtually every street and corner of the city—burning it openly, thereby, creating unpleasant smell and smoke throughout its neighborhoods. The collection of MSW in Benin City is not regular resulting in public health concerns (i.e., encouraging the spread of unpleasant and contagious disease), uncontrolled recycling of contaminated goods, pollution of water sources, and defacing of the city (Fig. 7)^25,26^. Benin City generates 573.6 tons of MSW daily—20% from plastic waste packed from hotels, restaurants, ceremonial venues, and public service centers, 50% from mixtures of organic and inorganic waste from urban and industry, and 30% from residential homes and public places^25,27^. The study area was purposively chosen as a representative city in Nigeria that faces a serious MSW problem in conjunction with a supporting entity (i.e., the EWMB) that has not been able to manage the growing waste influx.

### Research design and data collection

To explore the MSW problem in Benin City, a conceptual research design was put together based on categorized socioeconomic characteristics. A chi-squared test was used to calculate the difference between observed and expected values for each of the listed variables in Table 5. Totals for each of the categories was formulated using Eq. (1).

1 $${x}^{2 }=\sum\nolimits_{i=1}^{n}\frac{{({O}_{i}-{E}_{i })}^{2}}{{E}_{i}}$$

where ${O}_{i}$ = observed number of cases in category “$i$” and ${E}_{i}$ = expected number of cases in category “$i$”.

**Table 5:** Description of variables.

Variable	Description
Gender	Dummy variable: 0 = male, 1 = female
Age	Categorical variable: 1 ≤ 20, 2 = 21–35, 3 = 36–45, 4 = 46–55, 5 ≥ 56
Primary Occupation	Categorical variable: 1 = trader, 2 = farmer/fisherman, 3 = civil servant, 4 = private professional, 5 = other^a^
Education level	Categorical variable: 1 = GCE/SSCE/WAEC, 2 = NCE/ND, 3 = tertiary education
Private business	Dummy variable: 0 = not engaged, 1 = engaged
Marital status	Dummy variable: 0 = single, 1 = married, 3 = divorced, 4 = widowed
Awareness of GHG emissions effect on MSW	Categorical variable: 0 = not aware, 1 = aware

*GCE* General Certificate of Education, *SSCE* Senior Secondary Certificate Examination, *WAEC* West African Examination Council, *NCE* National Certificate of Education, *ND* National Diploma.

^a^Other = students, retired persons, and unemployed.

Data collection was based on a survey conducted by the Department of Environmental Sciences at the National Open University of Nigeria in Benin City from January to March of 2019. Survey oversight was conducted by the National Open University of Nigeria Ethical Committee in accordance with the guidelines and regulations as stated by the Declaration of Helsinki. Respondents volunteered to participate autonomously without their identity being recorded. The sampling process included all three of the predominant districts, i.e., Ikpoba Okha, Oredo, and Orhionmwon, and divided the city into four sampling zones: core, intermediate, suburban, and planned estates (i.e., new settlements). Blocking was applied in the design of the research using Eq. (2).

2 $${Y}_{ij}=\upmu +{T}_{i}+ {B}_{i}+e$$

where: ${Y}_{ij}$ equals the randomized block design for which sample area = $i$ and level of knowledge of GHG emissions = $j$; $\upmu$ is the mean; ${T}_{i}$ is the inclusivity for being in treatment $i$ in terms of the sample area; ${B}_{i}$ is the effect for being in block $j$ in terms of the of level of knowledge of GHG emissions; and $e$ is the random error.

The process of sampling used a cluster type technique to divide the city’s population into smaller areas. Random sampling was selected among these clusters to form the overall sample. Cluster sampling was the most appropriate method since it offered a probability sampling method often used in large population-based research^56–58^. The analysis arranged experimental units into groups to better illustrate variability in the knowledge of GHG emissions as well as MSW disposal among city residents. A binary matrix was employed to randomize the block design of the study area. Blocking and randomization were associated to the socioeconomic strata and place of residence—i.e., utilizing variables that bind or characterize a group collectively according to Wilk^59^. This approach helped to vividly illustrate the variation in the knowledge of GHG emissions as well as the danger embedded in indiscriminate waste disposal among the residents of the various residential areas of Benin City.

Data was collected from a total of 2720 randomly selected community members through a self-administered survey. Respondents were asked to rate perception and awareness levels using a four-point Likert scale (i.e., 1 = strongly agree, 2 = agree, 3 = disagree, and 4 = strongly disagree) (Table 6). Furthermore, direct domestic sewage disposal observational data was provided by the EWMB for the city, including: disposal methods, types of sewage generated, method of waste storage, and collection and disposal amounts.

**Table 6:** Key questions asked to respondents using a four-point Likert scale.

No	Question	SA	A	D	SD
1	Have you heard about the contribution of GHG emissions mitigation in the waste management sector?				
2	Are you aware of the implications of indiscriminate waste disposal to the environment?				
3	Are the GHG emission techniques and methods used by the government adequately creating awareness about the health implications of MSW in Benin City?				
4	Do you believe MSW disposal is growing towards a threatening level?				
5	Do you believe waste management can reduce the global effect of GHG emissions?				
6	Do you believe the level of urban waste control and management in Benin City is efficient?				
7	Did you know the government has formulated policies and measures to reduce GHG emissions?				
8	Do you believe the authorities responsible for the GHG emissions have sound knowledge of good practice?				

SA = strongly agree (1), A = agree (2), D = disagree (3), and SD = strongly disagree (4).

Descriptive statistics using SPSS version 20.0 was used to calculate mean, standard deviation, percentages, and cross tabulations (i.e., a chi-squared test and t-test) for data analyzation. Comparative findings using the Likert scale rankings was also performed using a chi-squared test (i.e., between groups). ArcGIS version 10.0 was used to produce overlay maps of the results.

It can be determined that economic progress brings more waste generation in Benin City, which by extension, is a general urban challenge in Nigeria. As a result, socioeconomic stratification can cause different perception and methods of waste disposal^60–62^. Ikolaba and Bodija, i.e., residents in the highest socioeconomic strata, have a better understanding of waste disposal as well as organized methods of disposing of it in comparison to Beere, Oje, and other low-income areas that practice indiscriminately dumping in Ibadan which is a very similar scenario in Benin City. These dumping methods bring different results to infant mortality with the former experiencing fewer infant deaths than the latter^60^. This national challenge parallels the low awareness of the contribution of MSW to GHG emissions and the direr, prospective health outlook for Benin City residents—corroborating findings throughout the country^18,35^. Since state capitals, such as Benin City, portray a fearfully low knowledge of the danger of indiscriminate dumping of MSW, it is not surprising that health implications become paramount^27^. During the fieldwork, qualitative inference of the situation noted that the rollout of radio jingles, especially in local parlance, could be used to appeal to the internal consciousness of the people to discourage indiscriminate dumping city-wide. It should also be noted that the observed changes in the local weather parameters throughout Benin City, despite the semi-national lockdown, further underscored other human related activities—including the regional MSW capacity which may also impact regional climate.

### Ethics approval

The National Open University of Nigeria Ethical Committee has approved that this study complies with the ethics of scientific research described in the National Open University of Nigeria Charter of Ethics and Ethical Principles of the Declaration of Helsinki and other applicable ethical principles and legislation in Nigeria. Ethical approval was granted by the NOUN Research Administration and Advancement, Research Ethics Sector, Ethical Committee.

### Consent to participate

Respondents volunteered to participate autonomously without their identity being recorded. Informed consent was obtained from all the participants in the study. Consent to participate was voluntary and approved by the National Open University of Nigeria Ethical Committee.

## Data Availability

All datasets are available from the corresponding author upon reasonable request.
